# Parental income gradients in adult health: a national cohort study

**DOI:** 10.1186/s12916-021-02022-4

**Published:** 2021-07-01

**Authors:** Miriam Evensen, Søren Toksvig Klitkou, Mette C. Tollånes, Simon Øverland, Torkild Hovde Lyngstad, Stein Emil Vollset, Jonas Minet Kinge

**Affiliations:** 1 Centre for Disease Burden, Norwegian Institute of Public Health, PO Box 222, Skøyen, 0213 Oslo, Norway; 2 Department of Inequality and Health, Norwegian Institute of Public Health, Oslo, Norway; 3 Norwegian Organization for Quality Improvement of Laboratory Examinations, Haraldsplass Deaconess Hospital, Bergen, Norway; 4 Department of Sociology and Human Geography, University of Oslo, Oslo, Norway; 5 Department of Health Metrics Sciences and Institute for Health Metrics and Evaluation, University of Washington, Seattle, USA; 6 Centre for Fertility and Health, Norwegian Institute of Public Health, Oslo, Norway

**Keywords:** Health inequalities, Childhood, Parental income, Cohort study

## Abstract

**Background:**

Disparities in health by adult income are well documented, but we know less about the childhood origins of health inequalities, and it remains unclear how the shape of the gradient varies across health conditions. This study examined the association between parental income in childhood and several measures of morbidity in adulthood.

**Methods:**

We used administrative data on seven complete Norwegian birth cohorts born in 1967–1973 (*N* = 429,886) to estimate the association between parental income from birth to age 18, obtained from tax records available from 1967, linked with administrative registries on health. Health measures, observed between ages 39 and 43, were taken from registry data on consultations at primary health care services based on diagnostic codes from the International Classification of Primary Care (ICPC-2) and hospitalizations and outpatient specialist consultations registered in the National Patient Registry (ICD-10).

**Results:**

Low parental income during childhood was associated with a higher risk of being diagnosed with several chronic and pain-related disorders, as well as hospitalization, but not overall primary health care use. Absolute differences were largest for disorders related to musculoskeletal pain, injuries, and depression (7–9 percentage point difference). There were also differences for chronic disorders such as hypertension (8%, CI 7.9–8.5 versus 4%, CI 4.1–4.7) and diabetes (3.2%, CI 3.0–3.4 versus 1.4%, CI 1.2–1.6). There was no difference in consultations related to respiratory disorders (20.9%, CI 20.4–21.5 versus 19.7%, CI 19.2–20.3). Childhood characteristics (parental education, low birth weight, and parental marital status) and own adult characteristics (education and income) explained a large share of the association.

**Conclusions:**

Children growing up at the bottom of the parental income distribution, compared to children in the top of the income distribution, had a two- to threefold increase in somatic and psychological disorders measured in adulthood. This shows that health inequalities by socioeconomic family background persist in a Scandinavian welfare-state context with universal access to health care.

**Supplementary Information:**

The online version contains supplementary material available at 10.1186/s12916-021-02022-4.

## Background

Individuals with low income tend to have worse health than those with higher incomes, and trends over the last decades reveal increasing health inequalities in many countries [1–6]. Strong socioeconomic gradients in health are also found in Scandinavia [7–9] despite universal access to publicly financed health services, free education, and strong welfare-state institutions [10]. Moreover, economic inequalities are also on the rise in Norway and other high-income countries—a development that may impact health inequalities both within and across generations [11, 12].

While a large body of research has documented the role of adult socioeconomic status in determining adult health [1, 3, 13], more recent research has focused on the importance of childhood factors for adult health. From these studies, evidence has accumulated that parental socioeconomic factors, childhood health, and residential context matters for later adult health and thus contribute to “the long arm of childhood” [14–16]. Yet, there are several shortcomings in the current literature.

First, most studies have focused on mortality outcomes. This line of research has shown that childhood socioeconomic disadvantage—typically measured by parental education, income, or occupation—is associated with higher overall mortality. More specifically, socioeconomic disadvantage has been linked with higher mortality related to heart disease, accidents, and alcohol and substance abuse [17–19], while the relationship with cancer is less pronounced [20]. Mortality in early adulthood is also higher among individuals who grew up in single-parent households and disadvantaged neighborhoods [14]. Adverse childhood conditions related to prenatal factors among low-SES children have also been found to predict mortality in early adulthood [21].

Although mortality differences are important, they represent a selected outcome since relatively few deaths occur in early adulthood or midlife [17, 22]. As most high-income countries face aging populations and longer life expectancies, there is also a need for more knowledge about the type of disorders for which health inequalities in early adult life are most salient, as most individuals will live long with these disorders.

Second, the existing research on morbidity in adulthood is primarily based on survey data, typically with small sample sizes, and few surveys provide reliable measures of parental socioeconomic resources throughout the entire childhood. Instead, several studies rely on retrospective accounts of childhood socioeconomic factors and often with only a few selected measures of health [23, 24]. Despite these limitations, this survey-based literature has linked low parental income, especially during early childhood, to increases in hypertension and arthritis measured in adult life [24]. Prior studies also linked low social origin to a higher risk of psychological problems, heightened levels of allostatic load [23, 25], and increased risk of poor cardiovascular health, substance abuse, musculoskeletal pain, and functional health [26–28]. In further advancing this topic, it is important to compare the association between parental income during childhood and adult health for a broad and comprehensive set of disorders as much of the current evidence relies on a selected subset of diagnoses or self-reported conditions. Moreover, there is a need for more knowledge on whether and how the strength and shape of this relationship vary across different disorders. Previous research has found that adult health increases monotonically with current income [3], but less is known about the shape of the association between parental income in childhood and health outcomes in adulthood. For instance, whether the relationship with parental income is linear or primarily manifests itself among those growing up in low-income families, with only weak associations between parental income and adult health above a given threshold.

Parental economic resources in childhood may influence adult health directly through prenatal and childhood health [29, 30] or indirectly through socioeconomic attainments and family formation in adulthood [31, 32]. For example, it is well established that children from low-income families obtain less education and lower earnings as adults, which constitute important determinants of health [33, 34]. However, differences in childhood health between children from less advantaged families, related to factors such as prenatal conditions, poor nutrition, or various stressors, may also contribute directly to the differences in adult health. There is less knowledge about whether upward mobility in education and the labor market attenuates the gradient in adult health by parental income or whether such gradients persist regardless of improved adult attainments. To examine whether the influence of childhood socioeconomic status primarily affects adult health through socioeconomic status in adulthood requires reliable data on individuals’ completed education and adult labor market outcomes. Further, few studies have compared these direct and indirect mechanisms for more than a few selected disorders.

This study addresses these issues by analyzing population-wide administrative data on parental income across the entire childhood and a wide range of morbidity measures based on diagnoses from general practitioners, specialist health care, and hospitalizations in adulthood for seven complete birth cohorts in Norway (*N* = 429,886). The data enable us to follow individuals from birth into adulthood, with annual reports on parental income up to age 18 and reliable data on health diagnoses an individual received between ages 39 and 43. We start by characterizing the shape and strength of the association between parental income and adult health outcomes for a range of disorders. Next, we evaluate the factors associated with parental income gradients in adult health using well-measured information on childhood context and own socioeconomic attainments and family formation in adulthood.

## Methods

### Data sources and study population

We used data from Norwegian administrative registries: the Central Population Registry, the National Registry for Personal Taxpayers, the National Education Database, the Medical Birth Registry (MFR), the Norwegian Control and Reimbursement Database (KUHR), and the National Patient Registry (NPR). Unique (de-identified) personal identifiers allow linkage between all registers and between children and parents. The parents’ annual income data was available from 1967, whereas health data was available for 2006–2016 for KUHR and 2008–2016 for NPR. We limit the study population to Norwegian birth cohorts born between 1967 and 1973, which allowed us to measure adult health outcomes at ages 39–43 consistently. Further, we restricted the sample to individuals who were alive and current residents in Norway by the latest follow-up, i.e., by age 43. An overview of the data sources appears in Supplement Figure S1.

### Measures

#### Parental income measure

Measures of parental income were constructed from annually reported pensionable labor market earnings, including wages and income from self-employment. Parental income was calculated as the sum of the biological mothers’ and fathers’ combined annual income averaged over the child’s age interval 0–18, with annual income adjusted to the 2016 Norwegian Consumer Price Index level.^1^ All individuals within each birth cohort were further ranked by childhood parental income and divided into vigintiles (i.e., 20 groups where each group represents 5% of all individuals). In the adjustment analysis (Fig. 3), we used a continuous measure of percentile ranks defined in the same way.

#### Adult health measures from data in primary and specialist health care

The Norwegian health care system is organized into primary and specialist health care. Primary health care comprises services such as consultations with primary care physicians and emergency room visits, while specialist health care includes somatic and psychiatric hospitals. All inhabitants who legally reside in Norway are members of the National Insurance Scheme and are assigned a general practitioner (GP). The GP must report diagnoses for each patient contact to the Norwegian Control and Reimbursement Database (KUHR), an electronic register available from 2006 onwards. These reports are the foundations for reimbursement and, therefore, unlikely to go unreported. Diagnostic information in this register is coded according to the second edition of the International Classification of Primary Care (ICPC-2) [35]. ICPC-2 contains codes for both diagnoses and symptoms of disorders. However, in the present study, we focus mainly on diagnoses.

We report results for adult health based on several measures of primary health care consultations. First, we measured the share of individuals who had one or more primary care consultations over a 5-year period.^2^ Second, we selected the 15 most frequently used codes for disorders (excluding symptom codes). In addition, we included consultations related to fear of specific diseases as an indicator of preventive and risk control behavior.^3^ The selected disorders were summarized into a single overall measure referred to as “any disorder.” For an overview of specific codes, see Supplement Table S1.

As a robustness check, we used data from the National Patient Registry (NPR), a nationwide registry covering patients in specialist health care, to classify somatic outpatient treatment, somatic inpatient treatment (hospitalizations), and psychiatric specialist treatment (inpatient and outpatient). Usually, a reference from the GP is necessary to get specialist treatment (except for acute hospitalization and treatment in emergency departments). Diagnoses in NPR are coded according to the 10th edition of the International Classification of Diseases and Related Health Problems (ICD-10).

#### Measures of childhood health, parental sociodemographic, and adult attainment

We report analyses stratified by selected measures of individuals’ childhood circumstances and adult attainments. To capture health at birth, we used a measure of low birthweight (infants who were below 2500 g at birth). Parental education was measured when the child was 16 years, by selecting the highest educational attainment level of either the mother or the father and categorized into three different levels: (1) less than upper secondary education (up to 11 years of completed education), (2) full secondary (12 years), and (3) tertiary (13 or more years). Marital status measures whether the mother was married when the child was aged 16.

To capture the individual’s own educational attainment in adulthood, we measured the highest level of completed education at age 35 in four categories: (1) below secondary (9 years), (2) full secondary (12 years), (3) tertiary short (13–14 years), and (4) tertiary long (15 years or more). Adult earnings were measured as the mean of own earnings averaged across the ages of 30–36 years and divided into quartiles. Measures of adult family status were constructed by combining information about marriage (married/not married) and parental status (have children/no children) measured by the latest follow-up (i.e., age 43). We differentiate between individuals who are or have been married and have children up to this age versus all others (i.e., no children and/or not married).

Finally, in some models, we control for childhood circumstance and adult attainments. In these models, we include all measures mentioned above and the mother’s age at birth, number of siblings, whether the individual was firstborn, and geographic region using municipality fixed effects measured at the child’s age 16.

### Statistical analysis

The primary aim of the analysis was to assess the association between parental income and a broad range of adult health diagnoses. For each adult health outcome, we estimate the following linear regression model:

1 $$ {H}_i={\beta}_0+{\beta}_1{Parental\ income}_i+{\mu}_i+{\varepsilon}_i $$

where *H*_*i*_ is the relevant health outcome of individual *i*, *Parental income*_*i*_ is the measure of parental income rank, and *μ*_*i*_ is a set of dummy variables for birth cohort. The parental income gradient in adult health is given by *β*_1_, which is the coefficient of parental income. To allow for non-linear gradients, we use a non-parametric specification where these gradients are from a set of dummy variables for parental income rank measured as vigintiles, and we report these results in a series of graphs.^4^

Our secondary aim is to assess whether controlling for a set of individuals’ childhood background factors (i.e., birth weight, parental sociodemographic characteristics, and the municipality of residence during adolescence) and adult attainments (i.e., educational attainment, adult earnings, and family status) accounts for the observed parental income gradients, similar to an approach used by Cutler and Lleras-Muney [36]. To achieve this, we first re-estimate Eq. (1) using a linear specification of parental income (i.e., 0 = bottom percentile, 1 = top percentile) that provides an overall estimate of the income-health association. Then, we estimate this equation while we add sets of covariates, ***Z***_*i*_, for individuals’ childhood characteristics and adult attainments:

2 $$ {H}_i={\alpha}_0+{\alpha}_1{Parental\ Income}_i+\delta {\boldsymbol{Z}}_i+{\mu}_i+{\varepsilon}_i. $$

Comparing the coefficients for parental income across different models with added covariates allows us to assess the percent reduction using the formula 1 − *α*_1_/*β*_1_.

All health outcomes are binary (i.e., 0 = no diagnosis, 1 = one or more consultations with a diagnosis). We present the predicted probability of a given diagnosis by parental income from linear probability models (LPM) estimated using OLS regression [37].

## Results

Table 1 shows that the study population consisted of 429,886 Norwegian individuals born in 1967–1973. Descriptive statistics are shown separately for individuals from the lowest childhood parental income decile versus everyone else, which reveal that individuals from the lowest income origins have a higher prevalence for most adult health measures; they grew up with less-educated parents and in less stable families. As adults, they had lower earnings, had less education, and were less likely to be married and have children. By contrast, overall health, which refers to whether the individuals have had at least one primary care consultation during a 5-year period, is similar across parental income groups.

**Table 1:** Descriptive statistics on adult health (age 39–43) and individual characteristics for Norwegian birth cohorts (1967–1973)

	All		Parental income, decile 1 (lowest)		Parental income, deciles 2-10 (highest)	
	Mean	95% CI	Mean	95% CI	Mean	95% CI, lower
**Measures from primary health care (KUHR)**						
Upper respiratory	0.204	[0.203, 0.206]	0.208	[0.204, 0.212]	0.204	[0.203, 0.205]
Acute sinusitis	0.119	[0.118, 0.120]	0.117	[0.114, 0.120]	0.119	[0.118, 0.120]
Back and neckpain	0.168	[0.167, 0.169]	0.199	[0.195, 0.203]	0.165	[0.164, 0.166]
Shoulder	0.139	[0.138, 0.140]	0.157	[0.154, 0.161]	0.137	[0.136, 0.138]
Bursitis	0.116	[0.115, 0.117]	0.124	[0.121, 0.127]	0.115	[0.114, 0.116]
Rheumatoid Arthrithis (RA)	0.013	[0.013, 0.014]	0.016	[0.015, 0.017]	0.013	[0.013, 0.013]
Hypertension	0.070	[0.070, 0.071]	0.081	[0.078, 0.084]	0.069	[0.069, 0.070]
Overweight	0.035	[0.035, 0.036]	0.042	[0.040, 0.044]	0.035	[0.034, 0.035]
Diabetes	0.024	[0.023, 0.024]	0.031	[0.030, 0.033]	0.023	[0.022, 0.023]
Gastroenteritis infection	0.010	[0.009, 0.010]	0.010	[0.009, 0.011]	0.009	[0.009, 0.010]
Depression	0.112	[0.111, 0.113]	0.142	[0.138, 0.145]	0.109	[0.108, 0.109]
Anxiety	0.040	[0.040, 0.041]	0.060	[0.058, 0.063]	0.038	[0.038, 0.039]
Fear of specific diseases	0.060	[0.059, 0.061]	0.064	[0.062, 0.066]	0.060	[0.059, 0.060]
Naevus (mole)	0.096	[0.095, 0.097]	0.083	[0.080, 0.085]	0.098	[0.097, 0.099]
Injuries	0.330	[0.328, 0.331]	0.359	[0.354, 0.363]	0.327	[0.325, 0.328]
Any disorder (any of above)	0.739	[0.737, 0.740]	0.767	[0.763, 0.771]	0.735	[0.734, 0.737]
Any conultation in primary care	0.955	[0.954, 0.955]	0.956	[0.954, 0.958]	0.954	[0.953, 0.955]
**Measures from specialist health care (NPR)**						
Outpatient	0.466	[0.465, 0.468]	0.506	[0.501, 0.510]	0.462	[0.460, 0.464]
Hospitalization, somatic	0.170	[0.169, 0.171]	0.199	[0.195, 0.203]	0.167	[0.165, 0.168]
Hospitalization, psychiatric	0.075	[0.074, 0.076]	0.113	[0.110, 0.116]	0.071	[0.070, 0.071]
**Individual measures from adulthood**						
Adult income (age 30-36, NOK 2016)						
Mean	298,326		257,773		302,832	
Std. dev.	364,183		115,977		381,663	
Education level (age 35)						
Below secondary	0.254		0.416		0.236	
Full secondary	0.369		0.362		0.370	
Tertiary, short	0.282		0.175		0.294	
Tertiary, long	0.091		0.034		0.097	
Missing	0.004		0.013		0.003	
Married with children	0.461		0.370		0.472	
**Individual measures from childhood**						
Parental income (age 0-18, NOK 2016)						
Mean	395,065		148,052		422,509	
Std. dev.	151,890		64,537		132,811	
Parental education (age 16)						
Less than full secondary	0.567		0.839		0.537	
Full secondary	0.195		0.098		0.256	
Tertiary	0.237		0.057		0.257	
Missing	0.001		0.057		0.006	
Mother not married at child age 16	0.154		0.365		0.131	
Mother's age at birth						
Mean	26.27		27.21		26.17	
Std. dev.	5.54		7.09		5.33	
Low birth weight	0.037		0.048		0.036	
Number of siblings						
Mean	1.690		1.720		1.690	
Std.dev	0.730		0.852		0.725	
First-born child of mother	0.705		0.711		0.705	
Municipality of residence at child age 16 (no.)	457		457		457	
Number of individuals	429,886		42,987		386,899	

### Childhood parental income and cause-specific diagnoses in adulthood

**Fig. 1:**
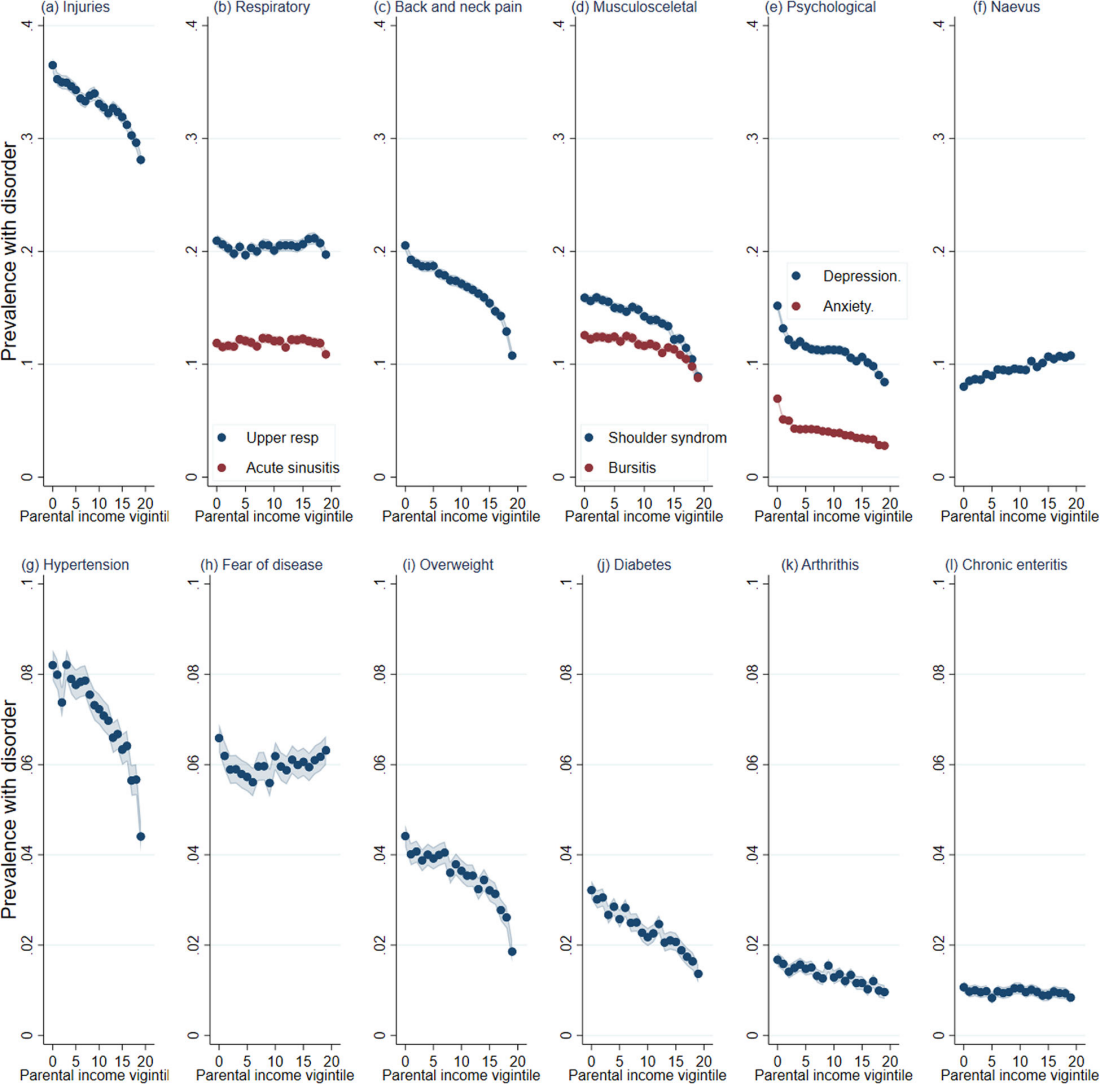
The association between parental income in childhood and adult health disorders in primary care, Norwegian birth cohorts 1967–1973. *Source*: data from the Norwegian Control and Reimbursement Database, 2006–2016. *Note*: predicted probabilities from linear probability models for childhood parental income vigintiles, controlling for birth year, estimated using OLS regression. Shaded areas refer to 95% confidence intervals. **A**–**L** The predicted probabilities of having one or more consultations for each specific disorder

Figure 1 shows the share with at least one diagnosed disorder (ages 39–43) by parental income vigintiles for the selected disorders (supplementary Table S2 report points estimates and 95% confidence intervals [CIs], Figure S3, panel C shows results for any disorder).^5^ The largest gap between individuals from the lowest and highest vigintile, in absolute terms, was found for injuries and back and neck pain. For example, 36.5% (CI 35.9–37.1) among those in the lowest income vigintile had an injury compared to 28.1% (CI 27.5–28.7) among those in the highest vigintile, a 9 percentage point difference. Similarly, 20.5% (CI 20.0–21.0) of individuals with parents in the lowest income had back and neck pain compared to 10.8% (CI 10.3–11.3) from the highest vigintile group; however, the shape of the gradient differed between the disorders. For injuries, the gradients were mostly linear, while the shape was less steep for back and neck pain. There was no difference between the parental income groups for respiratory disorders. Parental income was strongly correlated with psychological disorders. There was a 7 percentage point difference between those in the lowest (15.2%, CI 14.8–15.6) and those in the highest vigintile (8.4%, CI 8.0–8.9) for depression. The differences between those in the lowest vigintile (7%, CI 6.7–7.2) compared to the highest vigintile (2.8%, CI 2.5–3.0) were 4 percentage points for anxiety disorders. The shape of the gradient was non-linear, with considerably larger differences at the lowest levels of parental income compared to middle and high income.

For chronic disorders such as hypertension and diabetes, the gradients were, for the most part, linear. The gap between the lowest (8.2%, CI 7.9–8.5) and the highest (4.4%, CI 4.1–4.7) parental income vigintile was about 4 percentage points for hypertension. For diabetes and overweight, those in the bottom versus the top vigintile generally had a 2–3 percentage points higher probability of disorders. The overall prevalence of these disorders was low, ranging between 1 and 4%. For consultations related to naevus (mole) and fear, where fear of hypertension and breast cancer was the most frequent, there was an inverse gradient as higher parental income in childhood was associated with more consultations.

Women have more consultations than men, but the shape of the gradients is similar across sex (supplementary Figures S3, S4, and S5). The largest sex difference was found for injuries, with a weaker association across parental income for women than for men (supplementary Figure S4).

### Sociodemographic variation in the gradients of adult health by childhood parental income

**Fig. 2:**
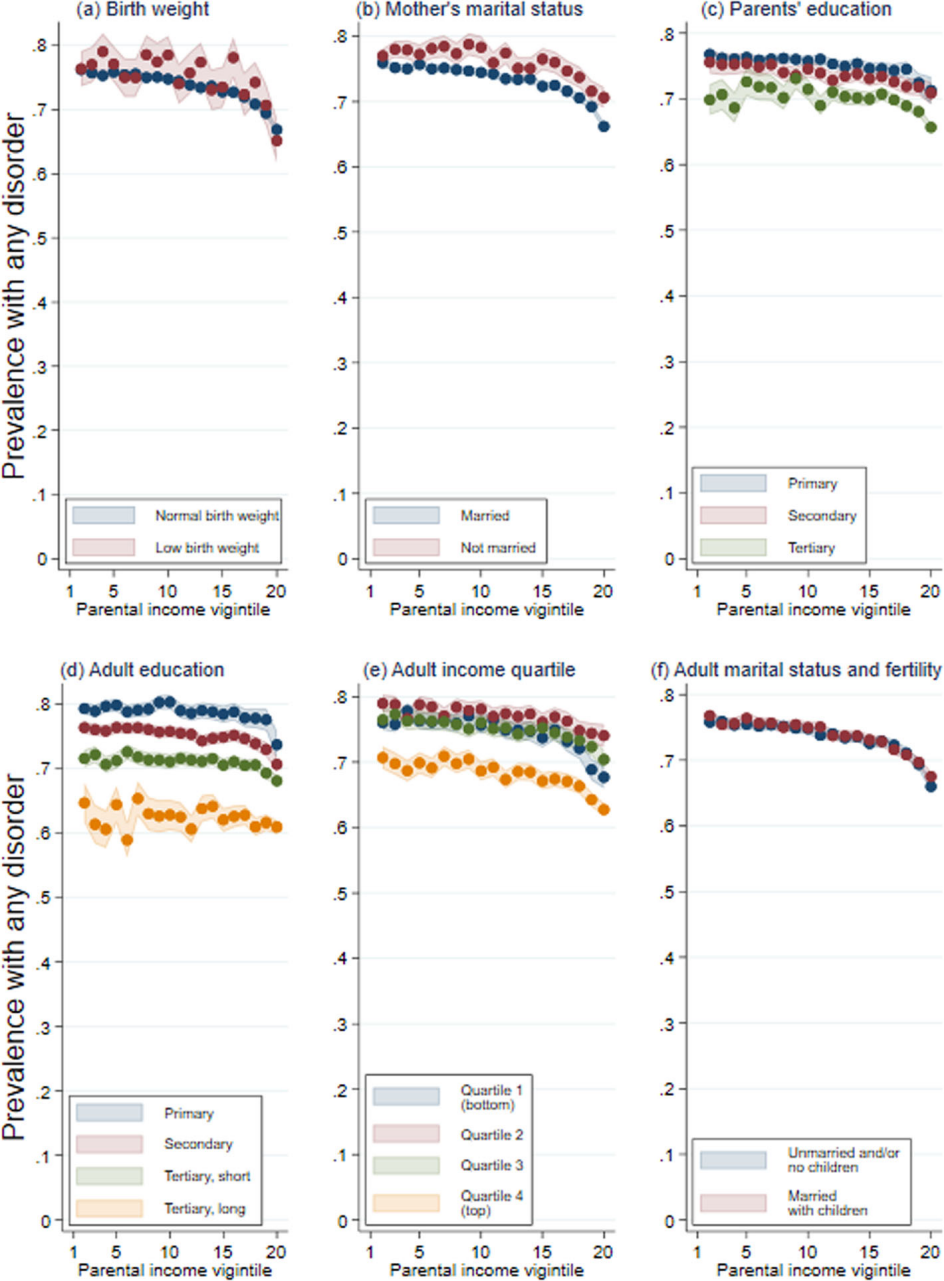
The association between parental income across childhood and adult health separately by childhood circumstances (**A**–**C**) and adult socioeconomic attainment (**D**–**F**), Norwegian birth cohorts 1967–1973. *Source*: data from the Norwegian Control and Reimbursement Database, 2006–2016. *Note*: predicted probabilities of having had one or more consultations for any disorder (i.e., one of the disorders in **A**–**L**) from linear probability models for childhood parental income vigintiles, controlling for birth year, estimated using OLS regression. Shaded areas refer to 95% confidence intervals. Each panel shows the predicted probabilities from regressions where each childhood parental income vigintile is separately interacted with dummy variable measures of birth weight (**A**), mother’s marital status at child age 16 (**B**), parental education at child age 16 (**C**), educational attainment at age 35 (**D**), adult income quartile averaged across ages 30–36 (**E**), and has been or being married with own children by age 43 (**F**)

Figure 2 presents results for the summary measure of any disorders stratified by childhood circumstances (panels A–C) and one’s own adult attainments (panels D–F). Panel A shows that the shape of the parental income gradient was similar within both groups of high and low birthweight children. There is also a parental income gradient in single-parent households and households with married parents (panel B). However, there is less evidence of a gradient within each level of parents’ educational level, although children of parents with primary and secondary education had a higher prevalence of disorders than those with tertiary level education (panel C). The lack of a gradient indicates that parental educational level explains part of the association.

Turning to the differences by own adult attainments, there is no gradient within each level of educational attainment (panel D). However, there are marked differences in the prevalence levels of any disorder by educational attainment. There are weak parental income gradients within all income quartiles for own adult income, except for individuals in the top quartile with the highest adult incomes (panel E). Nonetheless, those in the top adult income quartile have significantly better health than those found in other parts of the adult income distribution. Finally, there is a parental income gradient in health within both the group who are married with children of their own and the group who are unmarried and/or childless individuals (panel F).

### Childhood circumstances, adult attainments, and parental income gradients by disorder

**Fig. 3:**
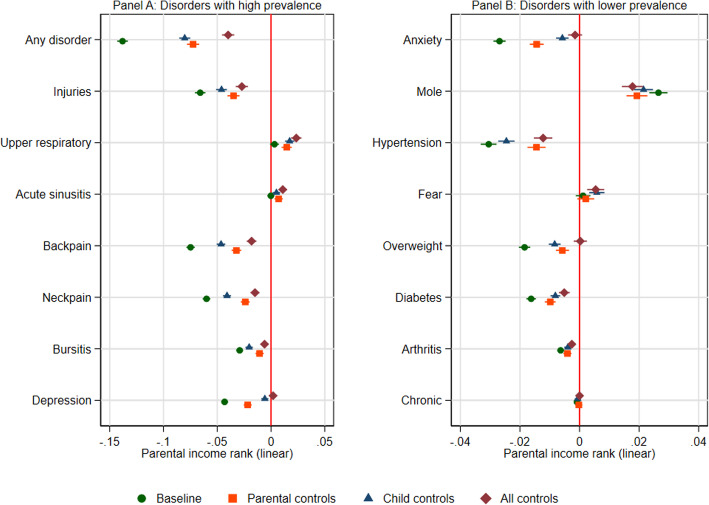
Association between parental income rank in percentiles during childhood and adult health outcomes, at ages 39–43, before and after adjustments for childhood factors and adult attainments for Norwegian birth cohorts 1967–1973. *Note*: results from linear probabilities model with 95% CI for linear specifications of the parental income gradients in adult health. Results are shown for (i) baseline model (i.e., controlling for birth year), (ii) models including controls for childhood factors (i.e., birth weight, mother’s age at birth, number of siblings, whether or not the child was the mother’s firstborn child, parental education, and municipality-of-residence fixed effects at child age 16), (iii) models including controls for adult attainments (i.e., education, income, and family status), and (iv) models including all control variables combined

Figure 3 shows results for (1) baseline linear specifications of the parental income gradients in adult health and the reduction after controlling for (2) childhood circumstances factors (i.e., birth weight, mother's age at birth, being firstborn, number of siblings, municipality of residence at child age 16 and parental educational levels; (3) individuals’ attainments in adulthood (i.e., education, income, and family status); and (4) all control variables. This allowed us to assess the extent to which the parental income gradients reflect differences in other childhood circumstances and adult attainments.

Out of 15 specific disorders, 10 were negatively related to parental income. All associations were severely reduced when adding controls. However, the negative relationship remained for most disorders. To be more specific, for our summary measure, the results show that adjustments for childhood circumstances reduce the parental income coefficient by roughly 50% while controlling for individuals own adult attainments reduce the coefficient by around 60% (see supplementary Table S4 for exact numbers). Adjusting for both sets of controls simultaneously reduces the coefficient by about 80%. The parental income gradients for most chronic and physical disorders are reduced by about 50–60% when controlling for additional childhood background factors, and similar reductions are found when adjusting for adult socioeconomic attainment. However, for psychological disorders such as anxiety and depression, adjusting for own adult socioeconomic attainment reduces the estimates by about 80–90% compared to about 50% when adjusting for childhood circumstances.

### Robustness check

One concern is that consultations in primary health care do not measure the most severe disorders and health conditions, although access to the more selective consultations in specialist health care usually goes through the referral of a general practitioner. To provide a robustness check of the results for primary health care service use, we report the results for somatic outpatient consultations, somatic inpatient consultations (hospitalizations), and psychiatric consultations (outpatient and inpatient consultations) in the specialist health care using data from the National Patient Registry.

**Fig. 4:**
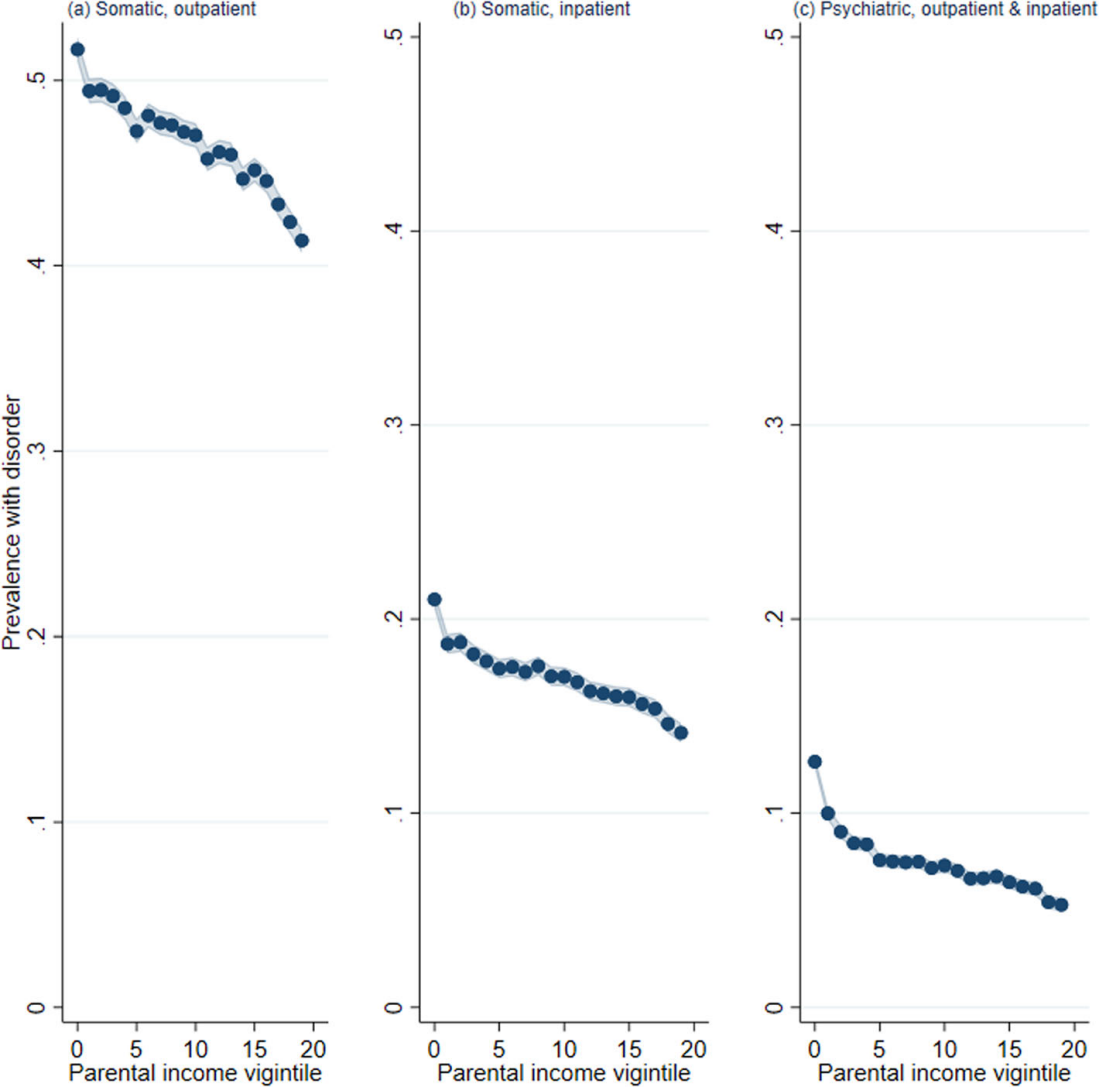
The association between parental income and health outcomes in specialist health care, Norwegian birth cohorts 1967–1973. *Source*: data from the National Patient Registry, 2008–2016. *Note*: predicted probabilities from linear probability models for childhood parental income vigintiles, controlling for birth year, estimated using OLS regression. Shaded areas refer to 95% confidence intervals. **A** The predicted probability of having had one or more somatic outpatient consultations in the specialist health care between ages 41 and43. **B** The predicted probability of having had one or more somatic inpatient (hospitalization) consultations in the specialist health care between ages 41 and 43. **C** The predicted probability of having had one or more psychiatric consultations (including outpatient and inpatient) in specialist health care between ages 41 and 43

Figure 4 reveals a similar pattern as reported for primary health care above, showing a relatively linear gradient where lower parental income is associated with higher probabilities of both outpatient and inpatient treatments of somatic conditions. For psychiatric treatments, the association strongly resembles the shape found in Fig. 1E for anxiety and depression with a more pronounced tail, with more than a twofold increased risk between individuals from the highest and lowest childhood income origins.

## Discussion

In this study, we used population-wide data to obtain estimates of social gradients in health for a broad range of disorders as registered in primary and specialist health care. Further, we identified the factors related to family resources and childhood upbringing that were correlated with social gradients in health. We found that parental income in childhood is related to adult health status for a wide range of disorders. The largest absolute difference was observed for disorders related to injuries and psychological and musculoskeletal disorders, with a doubling of the risk for most of these disorders between individuals who grew up in the bottom and top vigintiles of the parental income distribution. For hypertension, diabetes, and overweight, although less common in these age groups, there was a two- to threefold increase in disorders.

The documented health gradients are substantial and particularly so given that the study population was relatively young. Not only are these individuals expected to reach older age without their health intact, but these health differences may also have consequences for work capacity and retirement decisions [38, 39]. Our results corroborate studies with smaller sample sizes and less comprehensive data that document higher risks of disorders among individuals from low socioeconomic backgrounds for several chronic disorders, such as hypertension and diabetes, and psychological disorders. However, while prior studies primarily relied on self-reported measures [40], we examine a broader range of disorders using administrative data and a longer follow-up in adulthood than previous research.

Further, our findings provide nuance into the shape of the gradient across disorders as it documents possible threshold effects. While prior studies have documented that individuals from low social origins more often have musculoskeletal disorders in adulthood, we document that there were smaller differences up to the 15th percentile for such disorders. This indicates that these disorders are more equally distributed among lower and middle parental income origins but occur less frequently among those from higher-income origins. The gradient was steeper for disorders related to back and neck pain compared to pain-related disorders such as bursitis. It is likely that some of these associations result because of occupational exposures in adulthood, where heavy manual work is more related to back and neck pain consistent with prior research [41], while bursitis might be more related to tasks in white-collar occupations such as long durations of computer use. However, factors in childhood could also partially explain these associations. For example, prior research has also shown that childhood injuries, more frequently experienced by children with low parental background, relate to higher rates of self-reported chronic pain in adulthood [28].

The risk of injuries was higher among those with low income in childhood, consistent with previous research, which has found higher mortality due to accidents among disadvantaged families [17]. We found that it decreased continuously with higher parental income with little evidence for any threshold effects. However, injuries vary in type and severity, and we might speculate that non-linear patterns would be more apparent with a more fine-grained measure. For anxiety and depression, there was evidence of non-linear associations where the differences in health by parental income were considerably steeper among those with low parental income than among those whose families were in middle or high-income groups. The risk of experiencing mental health problems was thus disproportionately concentrated among persons with the most economically deprived family backgrounds.

These documented health gradients are unlikely to reflect a difference in access to medical care as we found, as have others [41], smaller differences in the overall share of primary care consultations between individuals from high- and low-income origins. However, although access to care is universal, the quality of care may differ by socioeconomic background. For consultations related to naevus (mole) and fear of specific diseases, we found an inverse relationship where higher childhood parental income was related to more consultations. This might indicate that individuals from high-income origins engage in more preventive and risk control behavior consistent with prior research showing socioeconomic gradients in, e.g., mammogram participation and medication adherence [42, 43].

Children who grow up in low-income families face disadvantages along several additional dimensions, such as prenatal health, family instability, and low parental education [31, 44, 45]. One of our key findings was that adult health gradients by childhood parental income were persistent within different subpopulations stratified by differences in childhood circumstances (i.e., marital status and birth weight), except for parental education. This implies that parental education and correlated unobserved traits (e.g., genetic endowments and parental health) may be underlying factors that partly confound the parental income gradient in adult health.

An important aim was to assess the contribution of additional factors of childhood context as well as potential mediating factors in early adulthood. We found that childhood circumstances, including parental education, account for about 50% of the parental income gradient. In addition, we explored some of the mechanisms through which the relationship between parental income and adult health status operates. For example, adjusting for individuals’ own education, income, and family status reduced the association by about 60% for most health disorders but as much as 90% for psychological disorders. However, problems of reverse causality might be important for all adult attainments, as individuals might fare less well as adults because of health problems rather than the reverse. Such a pattern of health selection may be particularly relevant with respect to the changes after adjustments we observe for psychological disorders.

There are strengths and limitations to the approach used here. Unlike most prior research, which uses data from self-reports or hospitalizations or mortality, we relied on physician-registered health disorders, a measure that complements other measures of health. However, the validity of accurate diagnoses is a concern, as research has shown that physicians differ in their evaluation of health problems [46–48]. For broader groups of disorders related to psychological and musculoskeletal problems, often with less specific symptoms, differences in physicians’ assessment may influence the results. Still, if there are systematic differences in the assessment of symptoms, our use of multiple health disorders should mitigate this bias. Our results are also limited to individuals who have been in contact with primary or secondary care services. If the tendency to seek medical help varies by childhood parental income, this may bias the association. Previous research has, for example, documented high rates of underdiagnosis for mental health conditions, even in countries with universal health care services [49, 50], and given the strong correlation between socioeconomic status and mental health, we may underestimate these associations. In contrast, our data do not cover private health care services. If the tendency to use the private sector is higher among high-income individuals, we might have overestimated the association. However, the use of private health care is low in Norway [10]. Finally, our sample was limited to persons who were alive and resided in Norway at the end of the observation period (i.e., at age 43). Higher mortality among persons from disadvantaged family backgrounds may lead to an underestimation of the parental income gradients in adult health, as deceased individuals, likely concentrated among those with the worst health, are not observed. However, any bias is likely to be modest, as deceased account for 3.7% of the birth cohorts covered. To what extent and in which direction outmigration may affect our results are uncertain. However, only 2.5% of non-deceased individuals in our birth cohorts lacked valid residential information at the end of the observation period.

Another caveat is related to the age of the sample. By focusing on health between ages 39 and 43, we likely capture some of the chronic diseases that begin to emerge and impair functional abilities, but we may also miss disorders with later onsets. For example, we found no relationship to respiratory disorders, which is surprising given the relation of income to smoking and physical activity [6]. However, smoking rates have drastically declined across generations implying that such factors may be less relevant for these and younger cohorts [51]. Instead, other worrisome trends have emerged, such as increases in childhood obesity [52] and higher rates of mental disorders among children and adolescents [53, 54]. How these trends will affect health disparities among the coming generations is a topic for future research. On many accounts, improved living standards and better public welfare-state services, such as high-quality childcare and universal health services, may have reduced adult health inequalities by socioeconomic family background. By contrast, increased rates of childhood poverty, a more ethnically diverse population reflecting recent immigration trends, and increased diversification of family forms (e.g., higher rates of divorce among low-income parents) may have resulted in stronger parental income gradients in health for children in more recent birth cohorts.

Finally, a limitation is that these estimates should not be interpreted as causal estimates. There could be selection effects, either through genetic or social transmission, that confound these associations. For example, parents’ poor health may both cause low parental income in childhood and poor health among offspring in adulthood, consistent with prior work on mortality, as well as other outcomes [55, 56]. Future research should focus on identifying the causal links and underlying mechanisms between childhood parental income and adult health.

## Conclusion

We found substantial associations between childhood parental income and adult health, particularly for injuries and psychological and musculoskeletal disorders. These disorders often have a recurring trajectory and have implications for the number of years lived in good health. Our study shows that there are important intergenerational socioeconomic inequalities in health within a society with universal access to health care services. Comparative research and focused evaluation of policy reforms are needed to assess the relative effectiveness of universal health care systems in reducing severe cases of ill health and promoting health equity regardless of family background.

## Supplementary Information

**Additional file 1: Table S1.** Codes for categorizing disorders. **Table S2.** Numerical values for Figure 2, 95% CI and coefficients. **Table S3.** Comparison of mothers’ union status by birth and at child age 16. **Table S4.** Baseline associations between parental income and disorders and reductions after adjustments. **Figure S1.** Study design and data sources, Norwegian birth cohorts 1967-1973. **Figure S2.** Comparison of measures of parental income before and after adjustment for household equivalence scales (EU and OECD). **Figure S3.** Share with any primary care consultation (panel A), distribution of diagnosis chapters (panel B) and share with any diagnosed disorders (panel C) by parental income vigintiles in childhood, Norwegian birth cohorts 1967-1973. **Figure S4.** Number of consultations across 5-years (panel A) and share with any primary care consultation (panel B) by parental income percentiles in childhood, Norwegian birth cohorts 1967-1973. **Figure S5.** The association between parental income in childhood and diagnosed disorders by each ICPC-2 chapter in primary care, Norwegian birth cohorts 1967-1973. **Figure S6.** The association between parental income in childhood and adult health disorders in primary care, Norwegian birth cohorts 1967-1973. **Figure S7.** Share with any disorder for separate measures of mother and father income rank in childhood (panel A) and share with any disorder separate by mother’s marital status (panel B). **Figure S8.** Unadjusted and adjusted association between parental income in percentiles in childhood and adult health (any disorder) at age 39-43, Norwegian birth cohorts 1967-1973.

## Data Availability

Data are not publicly available but can be accessed through Statistics Norway upon relevant approvals.

## Footnotes

1 Notice that we measure the income of both biological parents regardless of whether the parents are married or cohabiting or whether the child lives with both biological parents as it is likely that both biological parents’ income matter for children’s well-being.

2 Some of the individual registrations in KUHR does not necessarily reflect an in-person contact (they may be advice given by letter/phone/prescriptions/electronic contact, etc.) However, to measure disorders in this paper, we rely on diagnostic codes from face-to-face consultations based on tariff rates.

3 Codes related to fear of specific disorders are symptom codes. These codes refer to particular concern a patient may have related to symptoms of, e.g., breast cancer. The GP should refer the patient to a specialist, which will give a correct diagnosis.

4 To examine the sensitivity of our operationalization of the parental income measure, we further adjusted parental income for household size and composition, using equivalence scales. These adjustments had minimal impact on the results (see supplementary Figure S2).

5 The disorders are grouped by prevalence, where the most prevalent are presented on the top row with a common scale of 0.4. The less prevalent are grouped in the lower panel with a common scale of 0.1.
