# Supplementary material for: Parental income gradients in adult health: a national cohort study
Source: BMC Med. 2021 Jul 1;19:152. doi: 10.1186/s12916-021-02022-4 (PMC8247083; doi:10.1186/s12916-021-02022-4)
Supplement: Supplementary file 1 — Additional file 1: Table S1. Codes for categorizing disorders. Table S2. Numerical values for Figure 2, 95% CI and coefficients. Table S3. Comparison of mothers’ union status by birth and at child age 16. Table S4. Baseline associations between parental income and disorders and reductions after adjustments. Figure S1. Study design and data sources, Norwegian birth cohorts 1967-1973. Figure S2. Comparison of measures of parental income before and after adjustment for household equivalence scales (EU and OECD). Figure S3. Share with any primary care consultation (panel A), distribution of diagnosis chapters (panel B) and share with any diagnosed disorders (panel C) by parental income vigintiles in childhood, Norwegian birth cohorts 1967-1973. Figure S4. Number of consultations across 5-years (panel A) and share with any primary care consultation (panel B) by parental income percentiles in childhood, Norwegian birth cohorts 1967-1973. Figure S5. The association between parental income in childhood and diagnosed disorders by each ICPC-2 chapter in primary care, Norwegian birth cohorts 1967-1973. Figure S6. The association between parental income in childhood and adult health disorders in primary care, Norwegian birth cohorts 1967-1973. Figure S7. Share with any disorder for separate measures of mother and father income rank in childhood (panel A) and share with any disorder separate by mother’s marital status (panel B). Figure S8. Unadjusted and adjusted association between parental income in percentiles in childhood and adult health (any disorder) at age 39-43, Norwegian birth cohorts 1967-1973. [file 12916_2021_2022_MOESM1_ESM.docx]

Additional file 1: Supplementary Materials for Parental Income Gradients in Adult Health: A National Cohort Study

Authors: Miriam Evensen PhD, Søren Toksvig Klitkou, Mette C Tollånes, Simon Øverland, Torkild Hovde Lyngstad, Stein Emil Vollset and Jonas Minet Kinge

This file includes details on coding, construction of demographic and socioeconomic variables and sensitivity analyses and robustness checks, Tables S1-S4, Figure S1-S8.

**Supplementary Table S1**: Codes for categorizing disorders.

**Supplementary Table S2**: Numerical values for Figure 2, 95% CI and coefficients.

**Supplementary Table S3**: Comparison of mothers’ union status by birth and at child age 16.

**Supplementary Table S4**: Baseline associations between parental income and disorders and reductions after adjustments.

**Supplementary Figure S1**: Study design and data sources, Norwegian birth cohorts 1967-1973.

**Supplementary Figure S2**: Comparison of measures of parental income before and after adjustment for household equivalence scales (EU and OECD)

**Supplementary Figure S3**: Share with any primary care consultation (panel A), distribution of diagnosis chapters (panel B) and share with any diagnosed disorders (panel C) by parental income vigintiles in childhood, Norwegian birth cohorts 1967-1973.

**Supplementary Figure S4:** Number of consultations across 5-years (panel A) and share with any primary care consultation (panel B) by parental income percentiles in childhood, Norwegian birth cohorts 1967-1973.

**Supplementary Figure S5**: The association between parental income in childhood and diagnosed disorders by each ICPC-2 chapter in primary care, Norwegian birth cohorts 1967-1973.

**Supplementary Figure S6**: The association between parental income in childhood and adult health disorders in primary care, Norwegian birth cohorts 1967-1973

**Supplementary Figure S7**: Share with any disorder for separate measures of mother and father income rank in childhood (panel A) and share with any disorder separate by mother’s marital status (panel B).

**Supplementary Figure S8**: Unadjusted and adjusted association between parental income in percentiles in childhood and adult health (any disorder) at age 39-43, Norwegian birth cohorts 1967-1973.

**Figure S1:** Study design and data sources, Norwegian birth cohorts 1967-1973


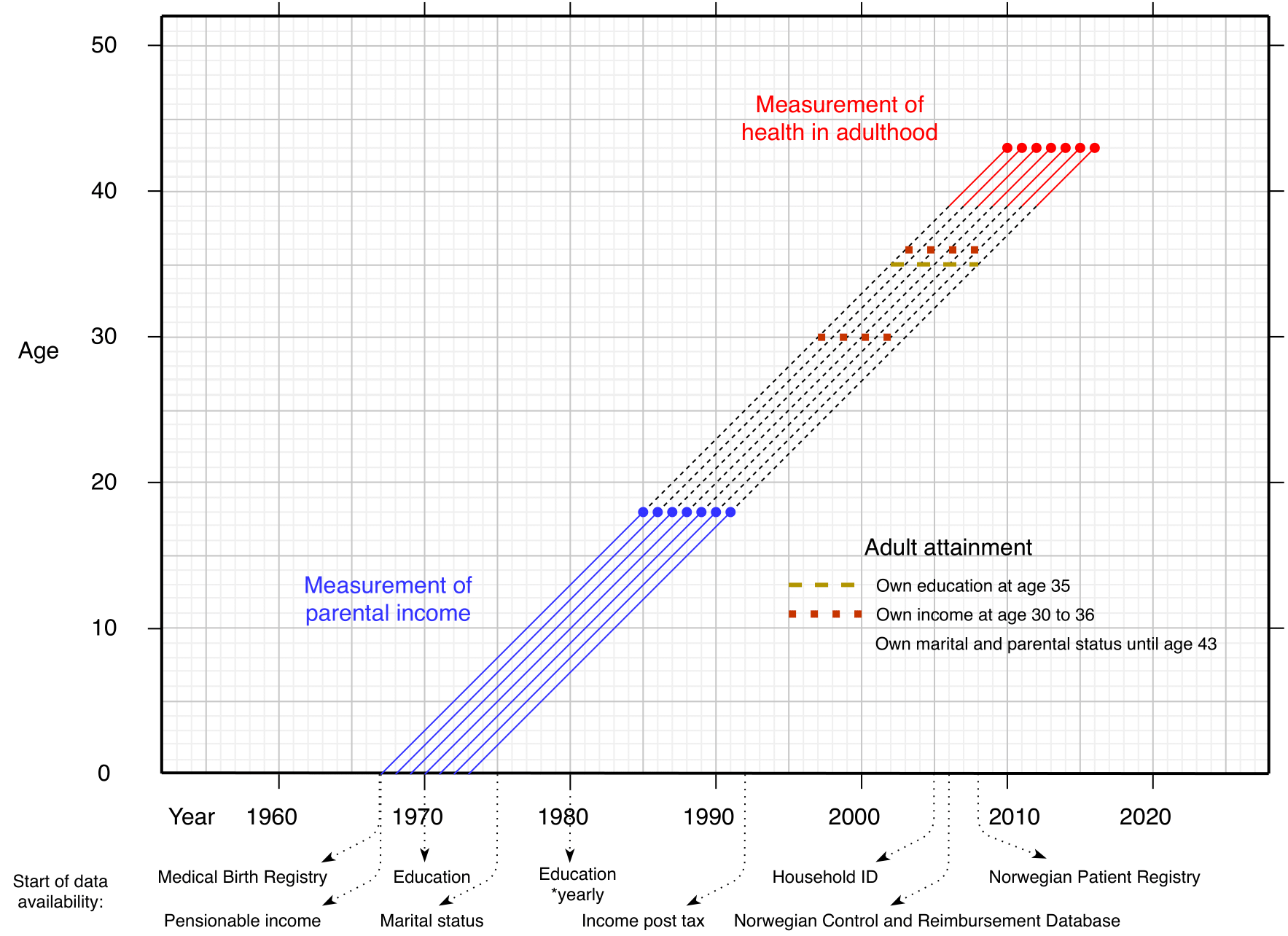


Table S1 provide an overview of the specific codes used from ICPC-2 and ICD-10.

Note: While most emergency units report injuries to the Norwegian registry for reimbursement of primary care providers (KUHR) some emergency care units report injuries to the National Patient Registry (57). We therefore included codes from ICPC-2 and ICD-10. First listed diagnosis was used.

**Comparison of measures of parental income before and after adjustment for household equivalence scales.**

In the main analyses, we rely on a measure of parental income that includes information on both parents’ income may regardless of parental union status and household size. However, household structure and size may be of importance. Registry data from Norway does not have detailed household information back to the early childhood period for children in our birth cohorts. Therefore, we cannot directly identify whether the child lived with both parents or with one parent and a new partner, as well as the number of co-resident children below age 18. Reliable household information is not available in these registries until 2005 and onwards. However, to get a better approximation of economic living standard, we conducted a sensitivity analyses where we use equivalized measures of parental income after imposing some (relatively strong) assumptions on the composition of households over time. We elaborate on this further below.

As union status was only available from 1975 in information from Statistics Norway and our birth cohorts are born between 1967-1973, we were able to get accurate information on marital status at birth by using information from the Medical Birth Registry (beginning in 1967). This means that we have reliable information about the mother's union status at birth and then annually from 1975 and onwards. Thus, if some individuals marry or get divorced (or widowed) between their child's birth year and 1975, we are unable to measure this. From 1975, however, we have annually updated information on whether the parent was married or divorced (but no information on cohabitating and exact household composition). Based on the available information, we, therefore, create two groups: For mothers who were unmarried at birth, we define a household as consisting of one adult and their children below age 18. For married persons, we define the household as consisting of the mother and father (i.e., two adults), plus common children below age 18. To adjust parental income household for size/composition, we used both the EU and OECD equivalence scale, such that the mother counts as 1, the partner 0.5/0.7, and each child 0.3/0.5, respectively. We show the results separately for all birth cohorts (1967-1973) and only those born in 1973, for which we have almost complete information on marital status.

Figure S2 shows that there is minimal difference between these measures, and these analyses highlight the robustness of several measures of parental income, albeit making strong assumptions about household composition.

**Figure S2:** Comparison of measures of parental income before and after adjustment for household equivalence scales (EU and OECD).

*
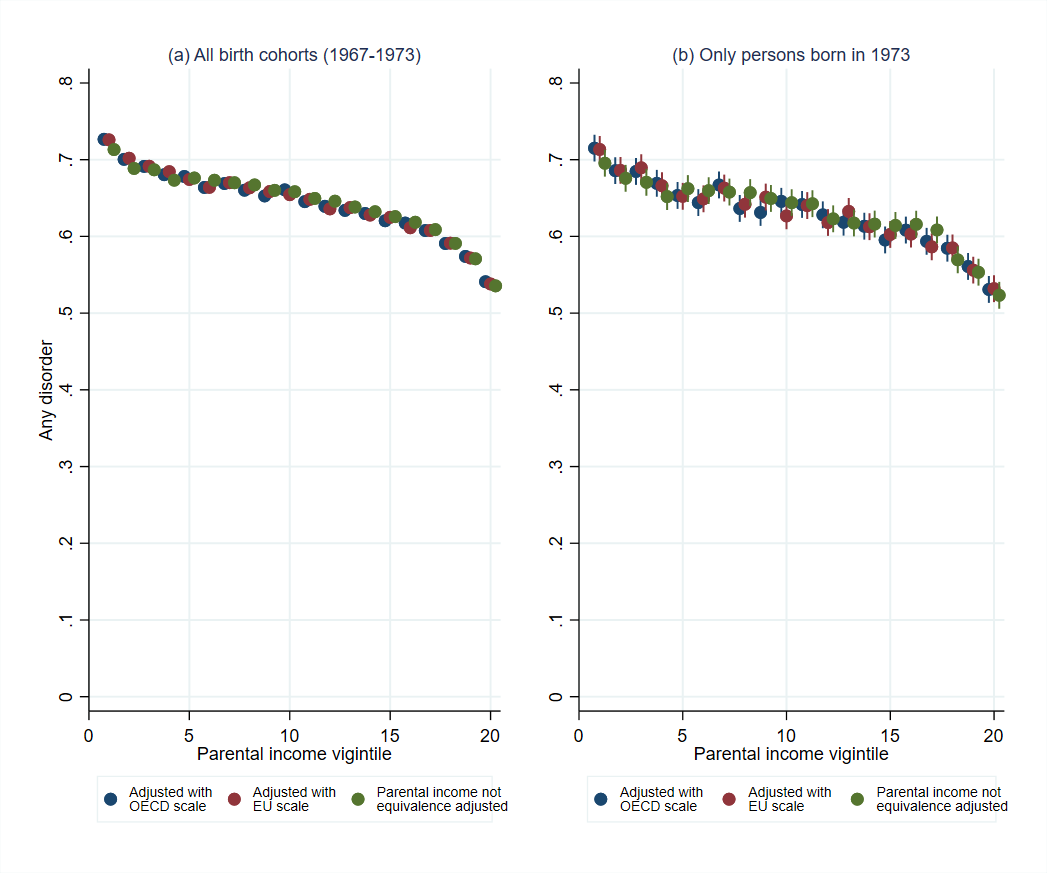
*

*Source*: Data from the Norwegian Control and Reimbursement Database, 2006-2016.

*Note*: Predicted probabilities from linear probability models for childhood parental income vigintiles, controlling for birth year, estimated using OLS regression. Shaded areas refer to 95% confidence intervals. To examine if the parental income measure was sensitive to operationalization, we adjusted parental income for household size and composition using equivalence scales. For mothers who were unmarried at birth, we define a household as consisting of one adult and their children below age 18. For married persons, we define the household as consisting of the mother and father (i.e., two adults), plus common children below age 18. We used both the EU and OECD equivalence scale, such that the mother counts as 1, the partner 0.5/0.7, and each child 0.3/0.5, respectively.

**Figure S3:** Share with any primary care consultation (panel A), distribution of diagnosis chapters (panel B) and share with any diagnosed disorders (panel C) by parental income vigintiles in childhood, Norwegian birth cohorts 1967-1973.


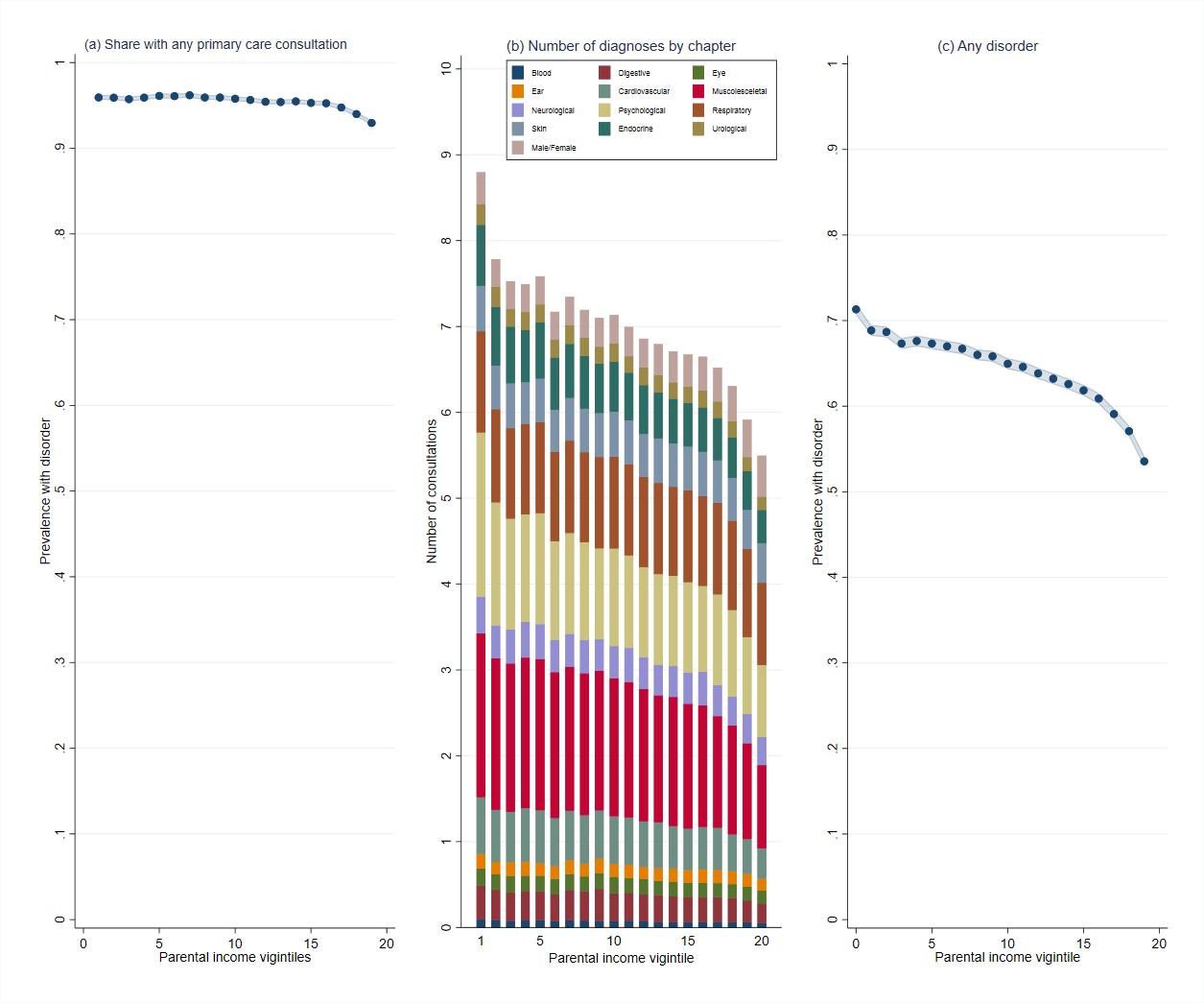


*Source*: Data from the Norwegian Control and Reimbursement Database, 2006-2016.

*Notes*: Childhood parental income vigintiles are averaged across the whole childhood, ages 0-18 years, and higher vigintiles refer to higher parental income. Panel A presents the share of individuals with one or more consultations in primary care in adulthood (ages 39-43) by childhood parental income vigintile. Panel B presents individuals’ average number of consultations in primary care for separate ICPC-2 chapters in adulthood (ages 39-43) by childhood parental income vigintile. We only include diagnoses within each category (excluding symptoms). Consultations related to Chapter A (General/unspecific) and W (Pregnancy-related disorders) are not included. Consultations from Chapters X (Female genital) and Y (Male genital) are combined (Wom/Men diag).

**Figure S4:** Number of consultations across 5-years (panel A) and share with any primary care consultation (panel B) by parental income percentiles in childhood, Norwegian birth cohorts 1967-1973.


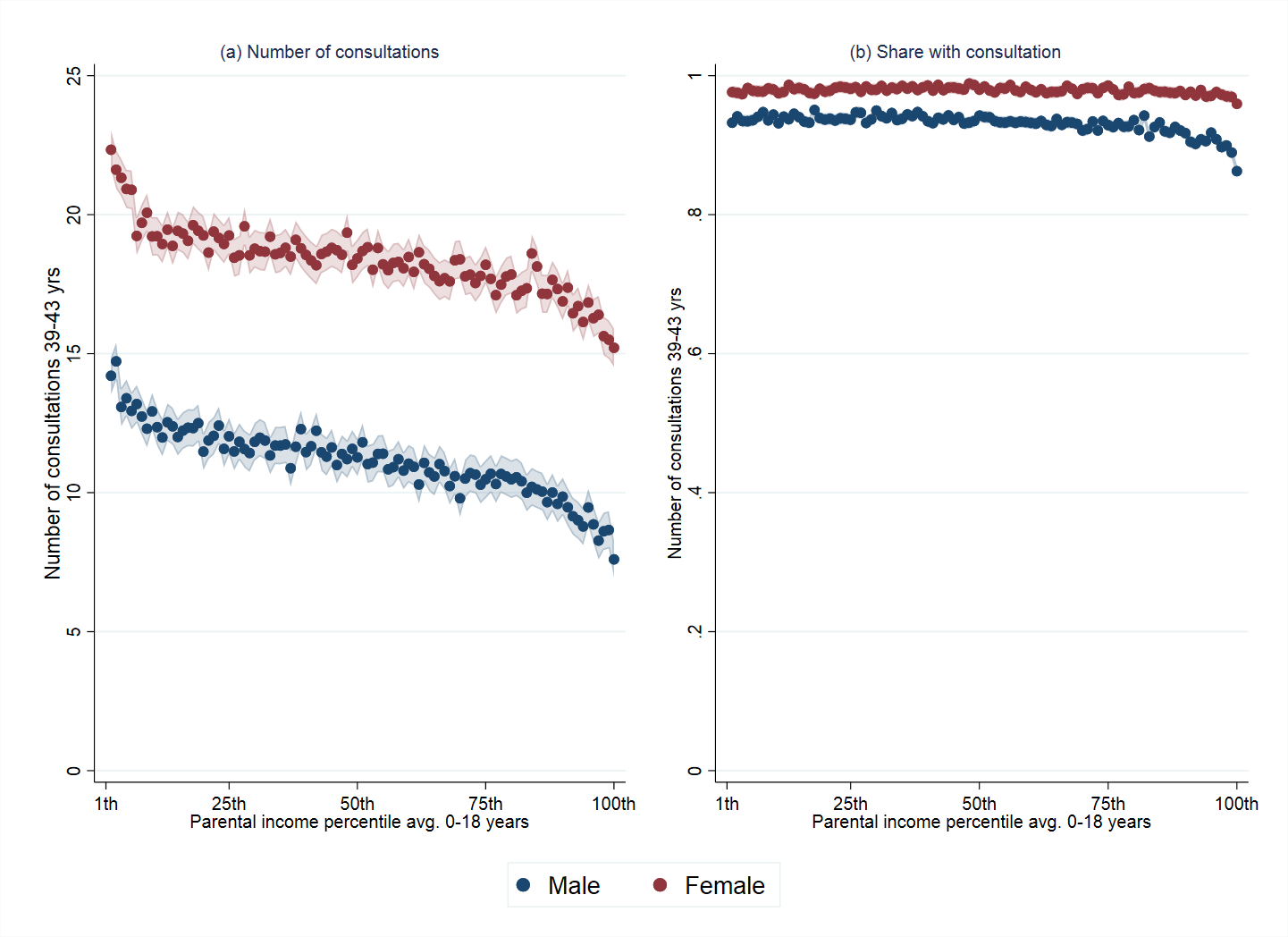


*Source*: Data from the Norwegian Control and Reimbursement Database, 2006-2016.

*Notes*: Childhood parental income percentiles are averaged across the whole childhood, ages 0-18 years, and higher percentiles refer to higher parental income. Panel A presents the average number of consultations in primary care in adulthood (ages 39-43). Panel B presents the share of individuals with one or more consultations in primary care in adulthood (ages 39-43) by childhood parental income percentile.

**Figure S5:** The association between parental income in childhood and diagnosed disorders by each ICPC-2 chapter in primary care, Norwegian birth cohorts 1967-1973.


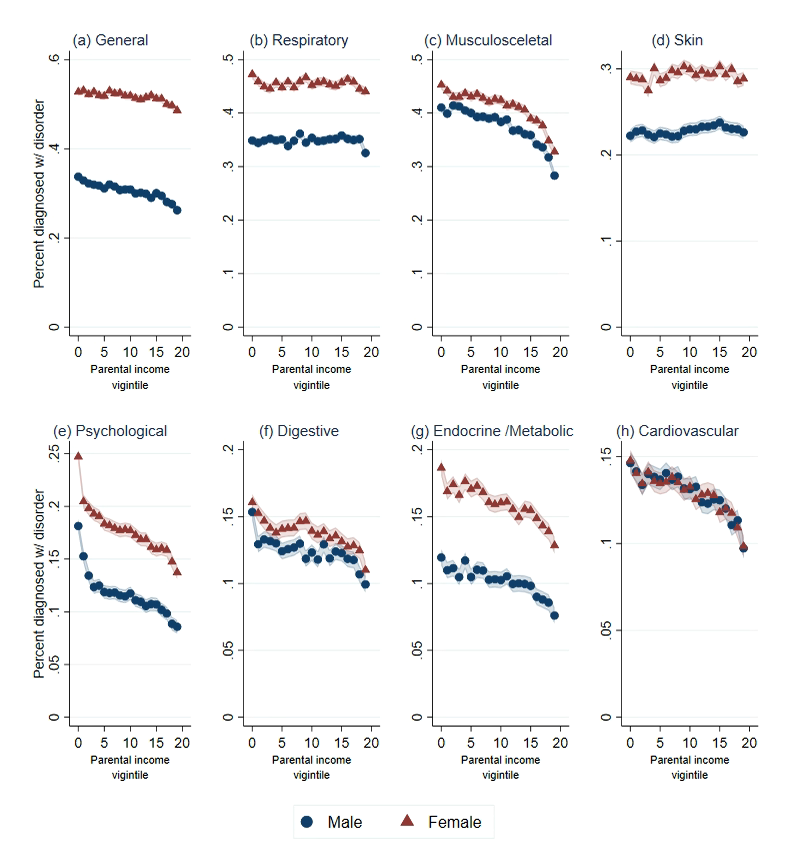


(continues on next page)


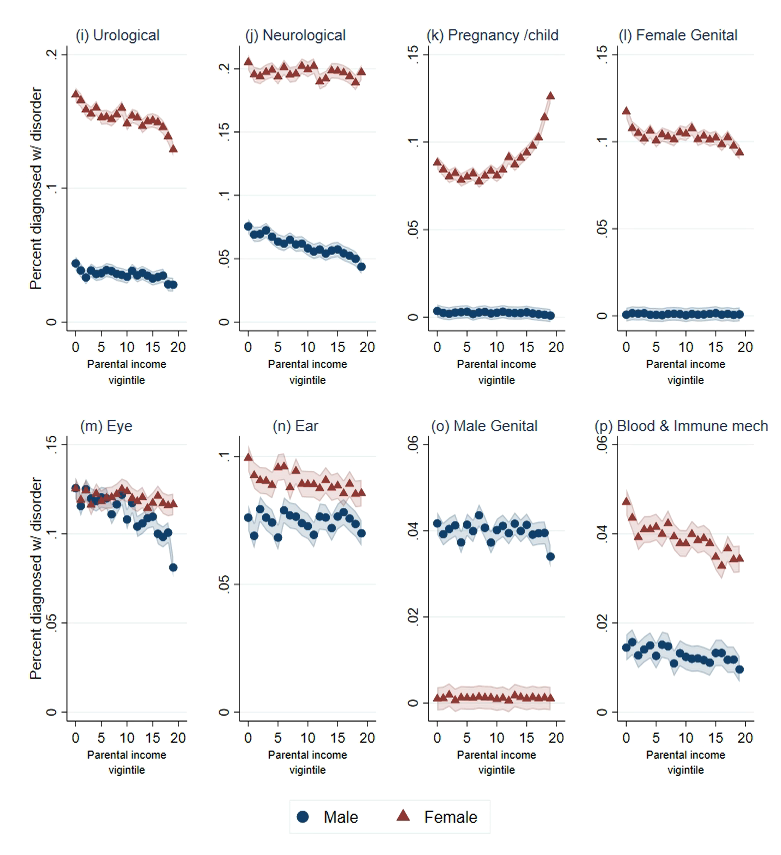


*Source*: Data from the Norwegian Control and Reimbursement Database, 2006-2016.

*Note*: Predicted probabilities from linear probability models for childhood parental income vigintiles, controlling for birth year, estimated using OLS regression. Shaded areas refer to 95% confidence intervals.

**Figure S6**: The association between parental income in childhood and adult health disorders in primary care, Norwegian birth cohorts 1967-1973.
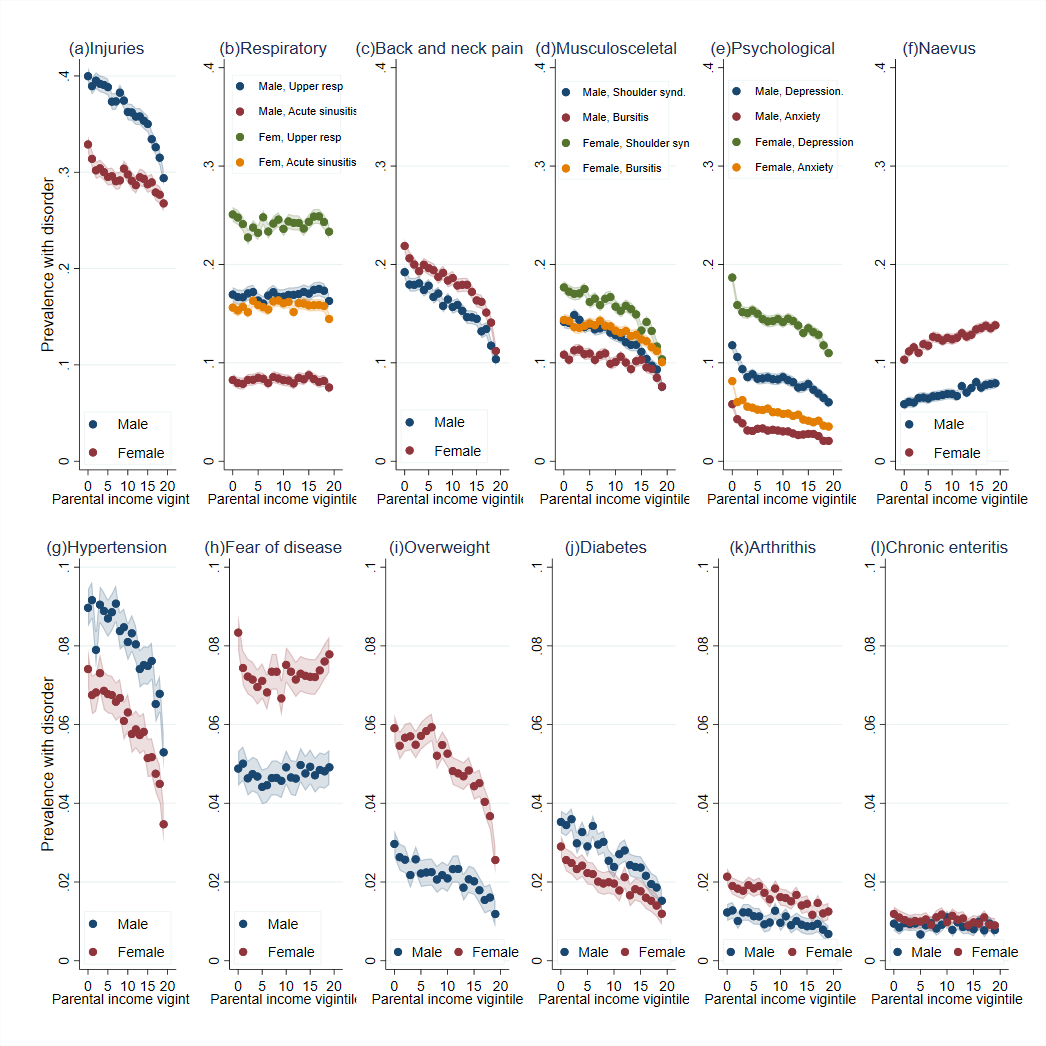


*Source*: Data from the Norwegian Control and Reimbursement Database, 2006-2016.

*Note*: Predicted probabilities from linear probability models for childhood parental income vigintiles, controlling for birth year, estimated using OLS regression. Shaded areas refer to 95% confidence intervals.

**Construction of demographic and socioeconomic variables and sensitivity analyses**

To construct covariates such as parental marital status and childhood residency, we used information from several registries from statistics Norway. Marital status is available in registries from 1975 and onwards, and we used information on the mother's marital status at the child age 16 to approximate the child's environment while growing up. Although these registries have information on marriage and divorce status, a drawback is that they do not have information on cohabitants. This means that there will likely be some misclassification of the non-married group as this group will consist of single and divorced mothers and mothers who have partnered but not married (cohabitants). To address the magnitude of this misclassification, we compared marriage rates at child age 16 to marriage rates at the birth of the child by using data from the Medical Birth Registry, see Table S3.

*Source:* Medical Birth Registry

A majority, 93 %, were registered as married by birth of the child; however, this share had fallen to 85 % when the child was 16. Given a higher share of non-intact families, one might wonder if the parental income gradient is primarily found among divorced families. We, therefore, performed a sensitivity analyses were we examined if the relationship between parental income and later health status similar for a subsample of families for which the mother report on being married at birth and where there is no record of the mother ever being divorced or deceased across child age 0-18.

Further, we speculated if the father's income was more strongly associated with worse health than mother's income. For example, the economic roles of mother and fathers for these cohorts are somewhat different from the typical dual breadwinner model. For some mothers, low income might truly reflect a disadvantaged position (i.e., indicate that they are low wage workers or have a marginal attachment to the labor market), however low income could also indicate that they were homemaking wives and thus reflect a "high class" position. Thus, mother's income might be a weaker signal of family resources for these cohorts as they are a mix of both high and low-status individuals. The results are found in Figure S7.

**Figure S7**: Share with any disorder for separate measures of mother and father income rank in childhood (panel A) and share with any disorder separate by mother’s marital status (panel B)


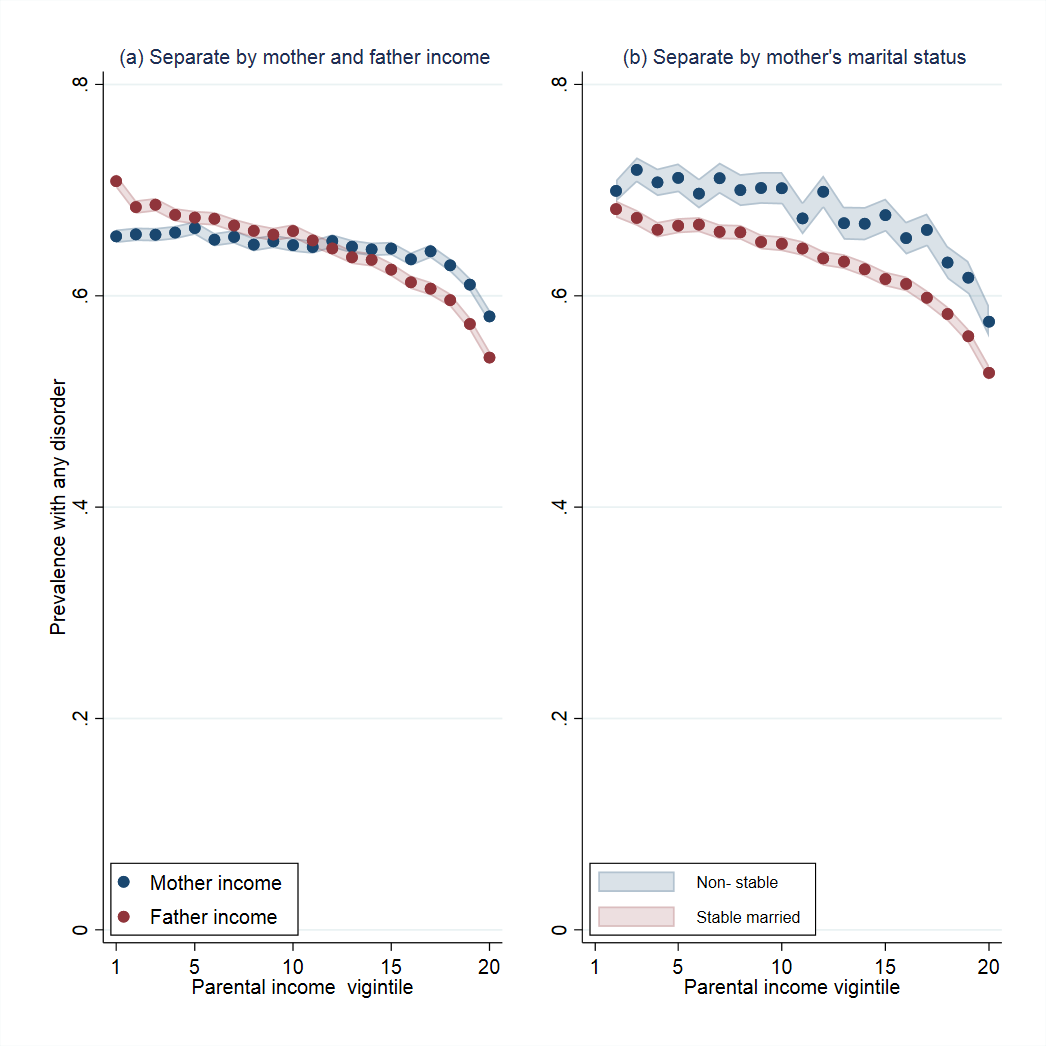


*Source:* Data from Norwegian Control and Reimbursement Database, 2006-2016

We found that father's income were more strongly associated with worse health at the tails of the distribution, while mothers income were more weakly related to any disorder. Based on the marital status, we found that, even though individuals of non-married mothers have a somewhat higher probability of disorders, the shape is similar for stable married and non-stable married samples, indicating that the gradient in health is not primarily found among non-intact families.

**Robustness of childhood circumstances and parental income gradients in adult health**

Figure S8 shows results for linear specifications of the parental income gradients measured in percentiles ranging from 0 to 1 (i.e., 0 = bottom percentile, 1= top percentile) in adult health and how the estimated coefficients decline after adjustments. First, we adjusted for childhood circumstances factors, such as birth weight, geographic region, mother’s age at birth and marital status separately, before adjusting for all childhood circumstances combined. Then, we adjusted for all adult attainment measures such as family formation, educational level and income separately before adjusting for all adult attainment combined. Lastly, we adjusted for all covariates combined.

**Figure S8**: Unadjusted and adjusted association between parental income in percentiles in childhood and adult health at age 39-43 (any disorder), Norwegian birth cohorts 1967-1973


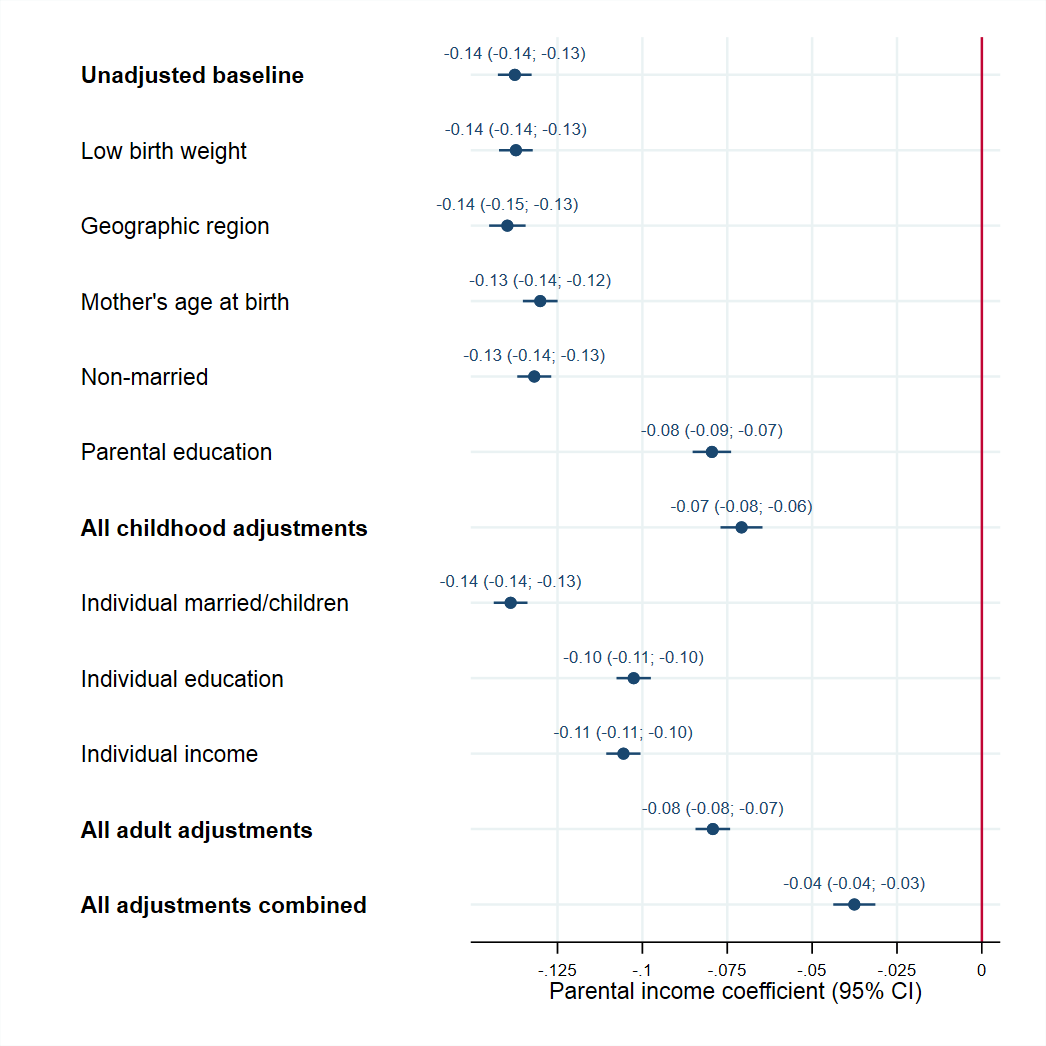


The results show that adjusting for birth weight, mother's marital status and geographic region (i.e., childhood place of residence) only reduces the parental income coefficient slightly. In contrast, parental education accounts for a larger part of the parental income association.
